# Super-Hydrophobicity of Polyester Fabrics Driven by Functional Sustainable Fluorine-Free Silane-Based Coatings

**DOI:** 10.3390/gels9020109

**Published:** 2023-01-27

**Authors:** Silvia Sfameni, Tim Lawnick, Giulia Rando, Annamaria Visco, Torsten Textor, Maria Rosaria Plutino

**Affiliations:** 1 Department of Engineering, University of Messina, Contrada di Dio, S. Agata, 98166 Messina, Italy; 2 Institute for the Study of Nanostructured Materials, ISMN–CNR, Palermo, c/o Department ChiBioFarAm, University of Messina, Viale F. Stagno d’Alcontres 31, 98166 Messina, Italy; 3 TEXOVERSUM School of Textiles, Reutlingen University, 72762 Reutlingen, Germany; 4 Department of ChiBioFarAm, University of Messina, Viale F. Stagno d’Alcontres 31, Vill. S. Agata, 98166 Messina, Italy; 5 Institute for Polymers, Composites and Biomaterials CNR IPCB, Via Paolo Gaifami 18, 9-95126 Catania, Italy

**Keywords:** sol-gel, alkoxysilanes, functional polyester fabrics, hydrophobicity, nano-hybrid coatings

## Abstract

Polyester fibers are widely employed in a multitude of sectors and applications from the technical textiles to everyday life thanks to their durability, strength, and flexibility. Despite these advantages, polyester lacks in dyeability, adhesion of coating, hydrophilicity, and it is characterized by a low wettability respect to natural fibers. On this regard, beyond the harmful hydrophobic textile finishings of polyester fabrics containing fluorine-compounds, and in order to avoid pre-treatments, such as laser irradiation to improve their surface properties, research is moving towards the development of fluorine-free and safer coatings. In this work, the (3-glycidyloxypropyl)trimethoxysilane (GPTMS) and various long alkyl-chain alkoxysilanes were employed for the fabrication in the presence of a catalyst of a water-based superhydrophobic finishing for polyester fabrics with a simple sol-gel, non-fluorinated, sustainable approach and the dip-pad-dry-cure method. The finished polyester fabrics surface properties were investigated by static and dynamic water repellency tests. Additionally, the resistance to common water-based liquids, abrasion resistance, moisture adsorption, and air permeability measurements were performed. Scanning electron microscopy was employed to examine the micro- and nano-morphology of the functionalized polyester fabrics surfaces. The obtained superhydrophobic finishings displayed high water-based stain resistance as well as good hydrophobicity after different cycles of abrasion.

## 1. Introduction

Polyester fiber textiles are popular among customers because of their durability, strength, flexibility, and resilience to climatic conditions, making them perfect for long-term usage in outdoor applications. Furthermore, the greatest benefit of polyester fibers is that they can be mass-produced in massive quantities at a low cost, resulting in lower costs for the customer as well [1,2,3]. On the contrary, polyester fabrics do have certain issues, such as a lack of breathability especially after some finishing treatments [4,5]. As a consequence, it does not absorb sweat from a person’s skin, retaining perspiration and heat adjacent to the skin, making people feel sticky and clammy, causing discomfort and bacterial growth. As a result, water repellent treatment for polyester fabrics is often required [6,7,8]. Furthermore, this can widen the breadth of polyester fabrics usage, increase its economic worth, fulfill the rising market request for more performant textiles, and improve their versatility. 

In order to increase the coating durability, it is crucial to increase the coating adhesion capability of polyester textiles themselves. In some investigations, polyester fibers were altered by UV irradiation, plasma pre-treatment or chemical hydrolysis to add functional groups to the surface, increasing the polymer’s hydrophilicity before the application of chemicals and functionalization [9,10]. 

As a matter of fact, fluorine and silicon finishing compounds are the most often used finishing agents [11,12]. In the past, despite their remarkable water repellent qualities, the most common industrial water-repellents contained perfluorooctanoic acid and perfluorooctane sulfonate, which were hard to breakdown and presented the risk of significant harmfulness and bioaccumulation [13,14,15]. For these reasons, their usage has been restricted, making critical the development of water repellents free of fluorine compounds. Nevertheless, there are a wide range of fluorine-containing formulations that can be utilized for improve the textile water repellency. Highly hydrophobic coatings are created from fluoroalkyl silane-based compounds and polytetrafluoroethylene-based ones [16,17,18]. 

Although opportunely coated fabrics can generate a water repellent surface with lower surface tension on the exterior of polyester fibers as in the case of silicone and polysiloxane water repellent agents, featuring the advantages of good biocompatibility and inexpensive raw material costs, the washing resistance is still insufficient [19,20]. 

Long-chain alkane compounds represent one of the existing water repellent finishing techniques able to introduce on cotton fabrics good water repellent properties. Unfortunately, due to the lack of functional groups able to bind to cotton fibers, they lead to the formation of finishings characterized by poor washing resistance, negative impact on hand feel, and poor air permeability [21,22].

One commonly explored strategy for producing superhydrophobic textiles is first to increase surface roughness by functionalizing the surface with proper inorganic precursors and nanoparticles via the sol-gel technique and then to reduce surface energy by attaching a hydrophobic groups [23]. Furthermore, the use of modified sols with suitable hydrophobic substituents may result in superhydrophobic and water-repellent films obtained in a single step [24,25,26,27,28]. Due to its adaptability, sustainability, versatility and simplicity, sol-gel chemistry is frequently used to produce functional and protective coatings for a wide range of industrial and applicative sectors, also allowing to obtain surfaces with improved mechanical strength, chemical stability, and thermal resistance [29,30,31,32].

**Figure 1:**
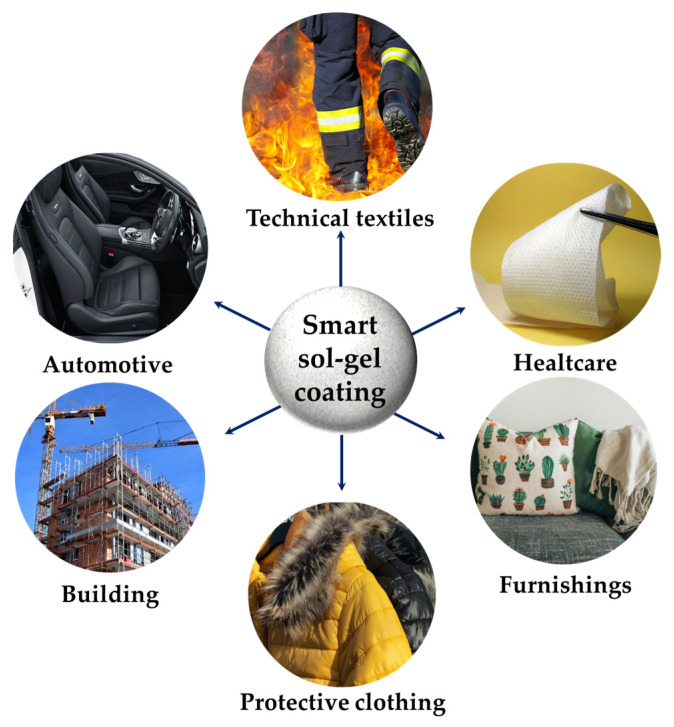
Potential application sectors of the purposed smart and sustainable non-fluorinated sol-gel approach to obtain superhydrophobic polyester fabrics.

Herein, in this study, a facile and eco-friendly sol-gel approach, to obtain superhydrophobic and breathable polyester fabrics, is presented; the chosen sol-gel reaction pathway is based on the hydrolysis and condensation of (3-glycidyloxypropyl)trimethoxysilane (GPTMS), crosslinked to different long alkyl-chain alkoxysilanes. The treated fabrics exhibited excellent superhydrophobicity useful for the assessment of their potential contribution to sustainable water repellent and fluorine-free textile coatings for a wide range of sectors, as shown in Figure 1 (i.e., from technical textiles and protective workwear to automotive, from buildings to biomedical applications, from furniture to high fashion industries). 

In particular, (3-glycidyloxypropyl)trimethoxysilane (G) was co-condensed with various non-fluoro substances, such as triethoxy(ethyl)silane (C2), triethoxy(octyl)silane (C8), and hexadecyltrimethoxysilane (C16), in the presence of HCl and 1-methylimidazole as catalysts for the GPTMS epoxy ring opening, to prepare nanohybrid cross-linked polyalkylsiloxanes coatings that were applied on polyester surfaces by the dip-pad-dry-cure deposition approach. 

The features of the finished polyester fabrics were additionally tested and examined by different chemical, physical, and mechanical techniques. In particular, the static and dynamic water repellency, resistance to conventional water-based liquids, abrasion resistance, moisture absorption, and air permeability of the coated polyester fabrics were analyzed, and the micro and nano-morphology of the functionalized fabrics surfaces were observed by scanning electron microscopy (SEM). Finally, a possible mechanism of the super hydrophobic behavior of the obtained micro- nano-structured hybrid coating was evaluated and proposed.

## 2. Results and Discussion

### 2.1. Water Repellent Nanohybrid Cross-Linked Polyalkylsiloxanes Sol-Gel Coating 

As for the cotton fiber substrates, water repellent finishing of polyester fabrics is often conducted using physical or chemical procedures that involve lowering surface energy and modifying the roughness and surface morphology of the fabrics specimens [33,34,35]. Unfortunately, although the many advantageous qualities of polyester, it has some disadvantages, such as poor dyeability, adhesion of coating, hydrophilicity, and low wettability in comparison to natural fibers, e.g., cotton, wool, and silk, due to its non-polar and hydrophobic character, which negatively affects the washing fastness of deposited finishings [36,37]. 

**Figure 2:**
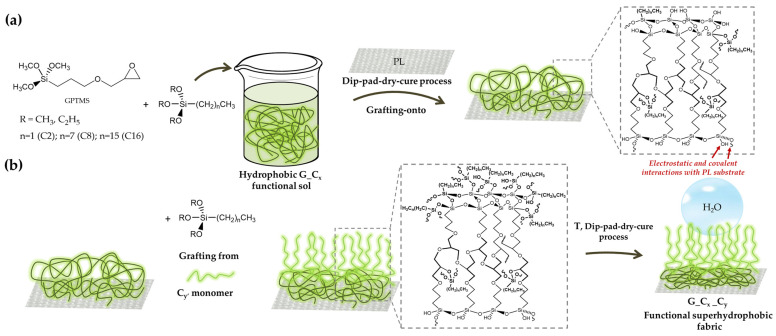
Description of the double surface treatment of polyester fabrics by “grafting to” or “grafting from” covalent functionalization techniques, involving condensation reactions between the substrate and the GPTMS/alkoxysilane xerogel (**a**), and subsequent linkage of the selected C2, C8 and C16 alkyl(trialkoxy)silanes (**b**).

In this study, in order to avoid surface pre-treatments, e.g., laser irradiation, the polyester samples (PL) were treated with the silane precursor (3-glycidyloxypropyl)trimethoxysilane as a crosslinker to encourage the formation of a tridimensional hybrid functional organic-inorganic network that displayed adhesive electrostatic and covalent interactions with the treated substrate. The development of these chemical contacts between the alkoxysilane precursors and fibers, however, can be aided by the presence of functional groups (hydroxyl and/or carboxyl) at the polyester polymeric ends [38]. Therefore, the establishment of covalent and electrostatic chemical connections between fibers and nanohybrid segments can be aided by these groups on the polyester surface (Figure 2a,b). In particular, a two-step pathway was performed in order to achieve the super hydrophobic coatings for polyester fabrics. 

In a first step, the bifunctional (3-glycidyloxypropyl)trimethoxysilane and one of the C2, C8, or C16 alkyl(trialkoxy)silanes, each characterized by an alkyl chain of a different length, were combined by a “grafting onto” approach to create the functional 3D polymeric matrix (Figure 2a) through a dip-pad-dry-cure procedure.

According to earlier reports [39,40], the functional sol-gel solution was developed by a subsequent hydrolysis and condensation reaction, which results in the dissolution of pre-condensed polymeric hybrid polymers or colloidal particles. The nanohybrid sol-gel will be converted into a nanostructured xerogel after being applied to polyester fabrics and after receiving extra heat treatment (at high temperatures). 

Five functional treated polyester specimens were produced by performing a second dip-pad-dry-cure step and using selected C2, C8, or C16 alkyl(trialkoxy)silanes, separately, in order to create more efficient hydrophobic finished polyester fabrics (Figure 2b). As a matter of fact, the in-situ graft density, structure, and molar ratio of the obtained brush polymer shells may be better controlled by a "grafting from" procedure. Moreover, this application technique leads to brush polymers that are not sterically constrained by the overall incoming functional long alkyl chains, thus leading to a notable decrease in surface energy and to the desired increase in hydrophobicity of the coated surfaces [41]. Additionally, it has been observed that the use of a double-layer deposition method can improve the properties, morphological features, and surface roughness of various developed films and coatings on textiles, thus resulting in improved mechanical and hydrophobic performances [40,42,43].

In this regard, WCA analysis was employed to study the static hydrophobicity of the functionalized polyester specimens. Moreover, spray tests were performed to determine their dynamic water repellence. 

#### 2.1.1. Aqueous Liquid Repellency Tests (Water/Alcohol Solution)

Using a reagent test, created in accordance with AATCC test method 193-2007, the contact angles of some aqueous liquids (characterized by various surface tensions) were measured to determine the degree of anti-wettability or repellency of the fabrics [44]. The water-rating method WRA, also known as the aqueous-liquid repellency test, is based on the application of 20 µL droplets of an isopropyl alcohol solution at various concentrations on fabrics (see Table 1). The following solution with a larger percentage of isopropanol is administered if the solution’s drops do not moisten the fabric within 10 s. The solution featuring the highest isopropanol content that doesn’t wet the fabric within 10 s is given the rating number. 

Table 1 summarizes aqueous repellency grade numbers, together with water/alcohol solution compositions and with related surface tension values. 

**Figure 3:**
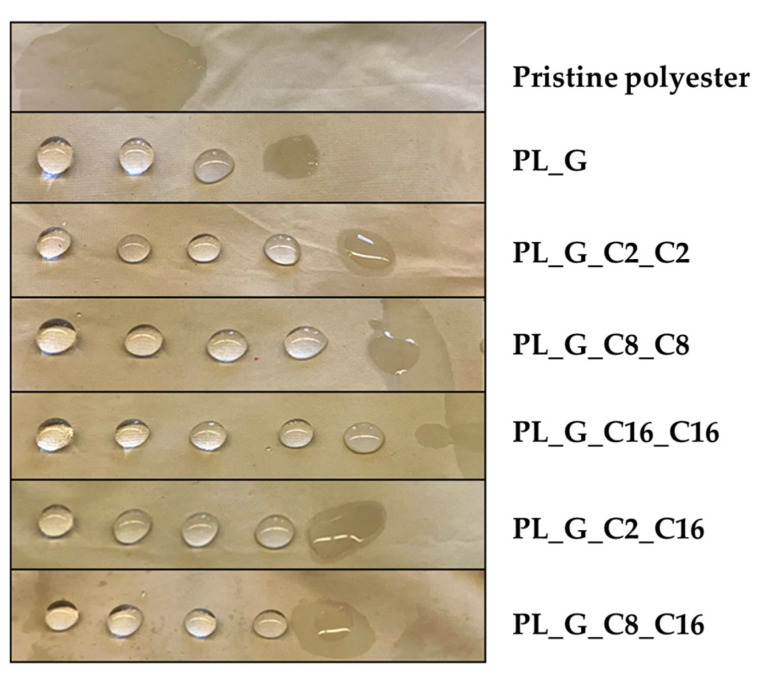
Static wettability test of the pristine and alkoxysilane modified polyester fabrics.

To be considered successful, the fabrics must be capable to reject the solution for 10 s (Figure 3). This technique uses solutions whose surface tensions are lower than those of water, resulting as a more rigorous static test than conventional water-drop techniques.

#### 2.1.2. Sessile Drop Method

**Figure 4:**
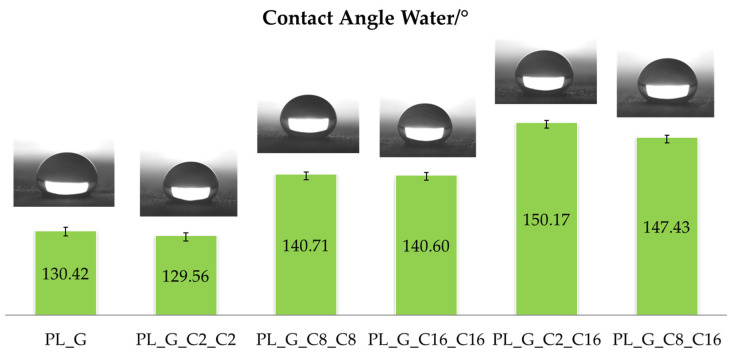
WCA value of different drops on coated polyester fabric and representative images captured with a video camera of typical static water drop tests for the various functionalized samples.

The sessile drop method is frequently used to directly measure the contact angle in order to test the wettability of specified coatings by distilled water [45]. In this regard, the hydrophobicity of the treated polyester fabric was examined and related to the length of the hydrocarbon chains of the functional alkyl(trialkoxy)silane being varied from 2 to 8 to 16 methylene groups. From an operational point of view, a deionized water droplet (about 5 µL) was gently deposited onto the surface in a normal procedure, and the photographs were taken with the help of a digital camera. To increase the measurement accuracy, all the water contact-angle values here provided were derived as averages of five measurements done on separate spots of the sample surface. The obtained results of the contact angle measurements reported in Figure 4 show that all the modified samples are strongly hydrophobic.

Furthermore, in the case of fabrics modified by combining short and long hydrocarbon chains, high WCA values, as those achieved for the samples PL_G_C2_C16 and PL_G_C8_C16, are obtained. In fact, by creating an asymmetric nanostructured hyperbranched polymeric coating with both long and short chain C2 or C8 and C16 co-monomers, it was able to replicate a brush effect that increased roughness while simultaneously reducing surface wettability [46]. The drop on the finished polyester fabric has a definite spherical form, as illustrated in Figure 4, and the best WCA is 150.17° for the specimen PL_G_C2_C16. However, after the impact on the untreated polyester surface, the liquid drop spreads out and is stretched quickly on the surface, still revealing good hydrophilic properties. 

Moreover, in order to compare the reported results with other published data, different examples of hydrophobic treatments for polyester fabrics and the relative WCA values are given in Table 2.

#### 2.1.3. Spray Test

**Figure 5:**
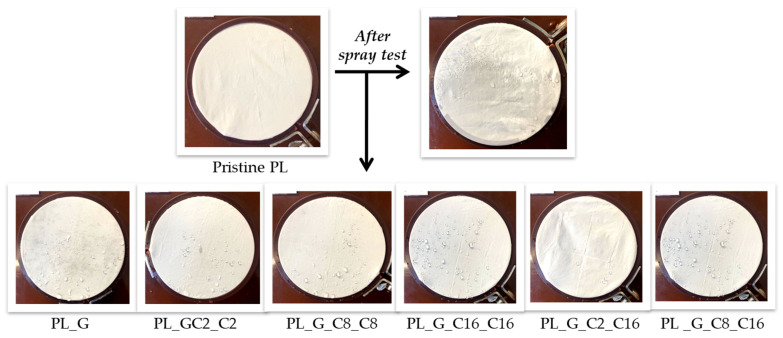
Spray test results for the dynamic wettability measurements of pristine and functionalized polyester fabrics.

The dynamic wettability of the treated polyester fabric was further investigated using spray testing (AATCC 22-2005) [53]. Spray testing measures the amount of moisture left on a fabric after being sprayed with water (Figure 5). Table 3 lists the water-stain characteristics (in ISO standard ratings) for various wetting degrees. The uncoated fabric’s wettability rating was 0, meaning it had no water repellency. As shown by the results, increasing the number of the alkyl chain improved the fabrics’ ability to repel water. After coating the sample PL_G, the functionalized fabrics wettability value climbed up to 50 and persisted at the same level also for the sample PL_G_C2_C2. Meanwhile, by increasing the number of the alkyl chain, the textiles’ ability to repel water could be improved, as demonstrated by the obtained results. In fact, spray testing revealed that the samples of treated polyester fabric PL_ G_C8_C8 and PL_G_C16_C16 had a rating number corresponding to 100.

Only a few droplets of the employed water volume (250 mL) that had been in touch with the treated textile samples remained quickly dispersed after that. The results show that polyester fabrics have high hydrophobicity and a low surface energy.

In contrast, the polyester fabrics exhibit greater surface energy when coated with GPTMS-based sol without the further addition of functional alkylsilane precursors. A water droplet placed upon the untreated polyester fabric spreads rapidly as well. 

#### 2.1.4. Self-Cleaning Behavior of the Fabric

**Figure 6:**
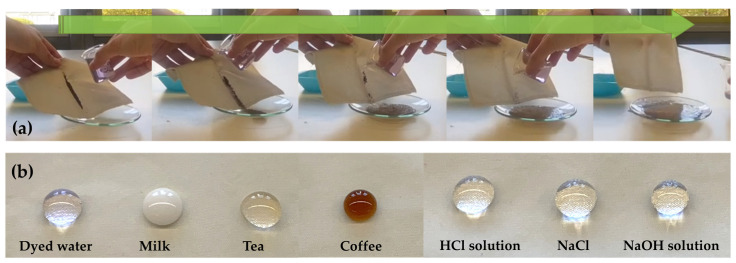
Self-cleaning illustration of coated polyester fabrics (A4 sample) by using wet soil as contaminant powder (**a**); a variety of common water-based liquids standing on the finished polyester fabrics (**b**).

The self-cleaning property of coated fabric (sample PL_G_C16_C16) is demonstrated in Figure 6. In particular, a certain amount of wet soil powder, used as model contaminants, was located on the functionalized specimen surface and then water was dripped from the impurity particles onto the fabrics surface as shown in Figure 6a. Rolling water droplets easily removed micron-sized contaminating particles from the fabrics, demonstrating the lotus effect of the functionalized polyester fabrics [54]. 

Additionally, as revealed in Figure 6b, some droplets of common liquids (i.e., dyed water, milk, tea, coffee, acid by HCl at pH = 1, alkali by NaOH at pH = 14) and salt solutions (NaCl, pH = 7) are able to assume the shape of a ball and remain on the surface of the polyester textiles treated with the different water repellent coatings, clearly revealing that they have got good stain resistance features to common water-based liquids.

### 2.2. Micro-Nano/Morphology Analysis by Scanning Electron Microscopy (SEM)

**Figure 7:**
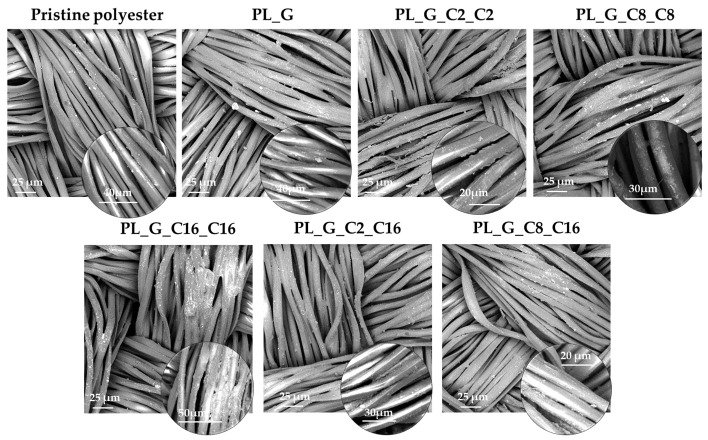
SEM images of untreated and functionalized polyester fabrics with the polyalkylsiloxane nanohybrid sols.

The micro-morphology of the water repellent fiber surface was observed by scanning electron microscopy. The surface morphology of the polyester fabric fibers, before and after water repellent coatings application, was studied after the gold coating of samples. The surface appearance of the fibers was detected and analyzed in order to study the relationship between the surface micro-morphology and water repellence features [55]. Figure 7 shows the micromorphology of pristine and treated polyester fabric surface after finishing. 

The results exhibited the rougher surface of the functionalized fabrics. The insets in SEM images of Figure 7 show at high magnification that the coatings accumulated on the fabric’s surface in a uniform manner. However, after the modification of polyalkilsiloxane-coated fabric, the surface morphology becomes inhomogeneous. As shown in Figure 7, the surface of the untreated polyester fibers is relatively smooth at low magnification with a uniformly distribution of the coating. Meanwhile, it can be clearly observed that there are many tiny grooves and many little protrusions on the surface of the treated polyester fabric fibers at high magnification which provide a nano-scaled roughness, supporting the superhydrophobic behavior of the treated fabrics [56]. 

A thin film is therefore covering the surface of polyester fibers consisting in the long alkyl-chain polysiloxanes grafted onto the GPTMS-based 3D matrix. In comparison with traditional fluoro-based materials, the developed functional and fluorine-free coating has higher water repellency, which can successfully reduce the surface energy of the polyester fabric specimen and improve the hydrophobicity of the fabric [57].

Microparticle aggregates coated the polyester fibers, thus significantly influencing the development of coated polyester fabrics with excellent hydrophobicity and water repellency.

### 2.3. Functionalized Fabric Performances

#### 2.3.1. Abrasion Resistance

**Figure 8:**
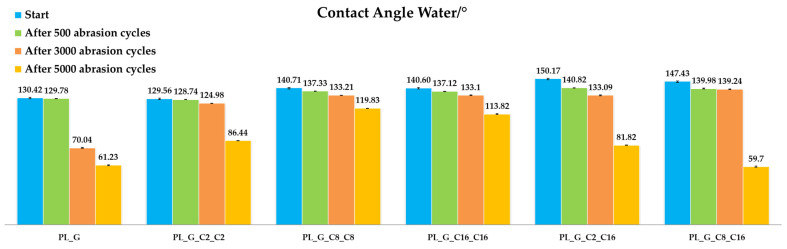
Static contact angle water measurements of the functionalized polyester fabrics before the abrasion tests and after 500, 3000 and 5000 cycles of abrasion.

Figure 8 illustrates the behavior of different nanohybrid coatings on polyester abrasion resistance, as evaluated through the Martindale method [58]. In all the experiments, some rises in the abrasion resistance of the treated specimens compared to pristine ones are evident. 

As the number of Martindale cycles increased up to 5000 cycles (end-point of the test because the control fabrics shows some defects), the wettability of the specimens gradually decreased. While this had no valuable effect on the general sample resistance, a more comprehensive analysis of the abrasion resistance behavior of treated textile supports displays that the appearance of the fabrics remains almost the same. In this regard, the quantity of catalyst is playing a key role. In fact, for polyester, the best behavior was achieved for the sample PL_G_C8_C8, showing a limited decrease of the static contact angle water even after 5000 cycles of abrasion.

#### 2.3.2. Moisture-Adsorption Analysis 

Moisture content has a crucial role in both cost and quality in a wide range of products, coatings and finishings. Compared to other synthetic fibers, polyester has a greater ability to avoid moisture absorption; this is one of the reasons why some types of polyester often tend to dry quickly [59,60]. Despite that, the presence of moisture also affects polyester fabrics, leading to harmful breakdown processes because the macromolecular chains hydrolyze, significantly reducing their molecular weight and material integrity [61,62]. However, it would be fair to specify that the permeability of a fabric largely depends more on its coating and its finish rather than only on the fiber with which it is made [63,64]. As a result, effective surface modification has a significant impact on how well materials absorb moisture. The moisture absorption characteristic of the developed coatings is reported in Table 4. As shown, the untreated polyester sample has a water absorption of 1.26%. Compared to the raw pristine fabric, from the data shown in the Table 4, it can be seen that the polyester fabric after finishing with the developed coatings presents a lower percentage of humidity, and this demonstrates once more how significantly the water repellency of polyester materials is improved.

The WVT values of the polyester fabric samples, treated with GPTMS, are lower than those of the original untreated samples. This water absorption was the same when using pure GPTMS-sol, since the modified GPTMS-based coating may still be hydrophilic. These GPTMS-sols must be changed with stronger hydrophobic components addition, such as hexadecyltriethoxysilane (a trialkoxysilane characterized by 16 carbon chain), to significantly alter their reaction to water absorption. The addition of the alkyl(trialkoxy)silane monomers therefore intensifies this decrease in proportion to the lengthening of the alkyl(trialkoxy)chain.

#### 2.3.3. Air Permeability Test 

**Figure 9:**
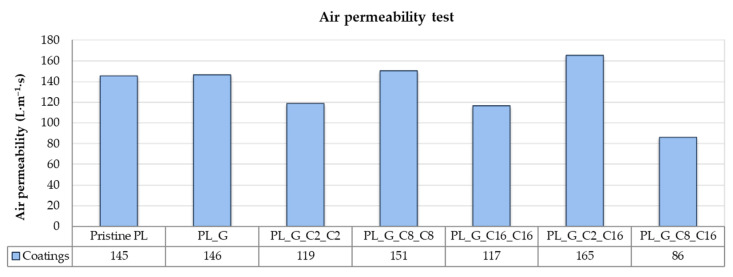
Air permeability results for the different obtained uncoated and coated samples.

Figure 9 summarizes the results of testing the breathability of coated polyester fabric and pristine polyester fabric in terms of air permeability. One of a fabric’s most crucial properties, primarily intended for technological or smart textile applications, is air permeability, which is closely related to porosity [65]. Finishings can influence this behavior because it is apparent that non-coated fabrics exhibit higher permeability than treated fabrics because of their low thicknesses and high porosity [66]. Here, it is clear that the air permeability of fabrics is slightly reduced by treating fabrics with hydrophobic alkyl(trialkoxy) silane formulations. For the PL_G_C16_C16 and PL_G_C8_C16 samples, the air permeability of polyester fabrics was not significantly impacted by the coating, indicating good overall breathability of the textiles substrates and making it appropriate for use in a variety of industrial-related areas. 

## 3. Conclusions

The treatment of polyester fabrics, using first a hybrid sol prepared by hydrolysis and condensation of GPTMS under acidic condition and in the presence of 1-methylimidazole as catalyst and long chain alkoxysilanes, and then hydrolyzed long chain alkoxysilanes, imparts a super-hydrophobic behavior to the coated polyester textiles. The results of the wettability test, as evidenced by the overall experimental data, led us to conclude that the addition of long alkyl-chain alkoxysilanes resulted in rougher surfaces, which decreased the surface free energy of the functionalized fabrics. As a matter of fact, the better alignment of the different long alkyl chains on the coated polyester surface and the increase in the textiles surface roughness are two explanations for the better featured water repellency. Furthermore, the treatment of the polyester fabrics causes a limited reduction in moisture adsorption and air permeability. Moreover, the treated polyester fabrics retain most of the hydrophobic properties even after 5000 abrasion cycles. The proposed two-step coatings system highlights that the use of fluorine-containing compounds is not strictly necessary and it can be easily replicated and extended towards a more sustainable preparation of transparent water-repellent functionalized surfaces over a large area. Last but not least, it may also offer a convenient alternative to traditional methods for the production of hydrophobic and superhydrophobic textile fabrics. Furthermore, in the case of polyester surfaces, this method has been shown to be an efficient and stable way to obtain functional coated fabrics, even in the absence of conventional polyester chemical and physical pre-treatments.

The obtained good water repellency, shown by the developed coatings, led us to conclude that this environmentally friendly, cost-effective, and relatively simple synthetic procedure may have positive effects on the production of a wide variety of textile fabrics featuring extremely high hydrophobicity and water-based anti-stain performance.

## 4. Materials and Methods

### 4.1. Materials

A commercial plain weave PET fabric (multifilament) provided by TEXOVERSUM School of Textiles (Reutlingen University, Reutlingen, Germany) was used for the experiments. The fabric has mass per unit area of 65.5 g/sqm, a thickness of 0.1 mm and a yarn fine of 5.1 tex (tex = g/km) (the average filament diameter is 11 µm). The warp and weft density are 50/cm and 75/cm respectively. Deionized water was employed in all the experiments. Highest purity level (3-glycidyloxypropyl)trimethoxysilane (G), hexadecyltrimethoxysilane (C16), triethoxy(octyl)silane (C8) and triethoxy(ethyl)silane (C2) were purchased from Merck Co. (Darmstadt, Germany) and employed without further purification. Hydrochloric acid (HCl 37% wt.) and 1-methylimidazole were used as sol-gel and GPTMS epoxy ring opening catalysts respectively. Ethanol (96% vol.) and isopropanol were provided from Sigma Aldrich. 

### 4.2. Nanohybrid Functional Sols Synthesis

Three distinct alkoxysilanes (C2, C8, and C16) with progressively longer hydrocarbon chains were separately employed and combined with an equimolar amount of the G precursor (1 mol) to create the desired functional final sol-gel solution. Ethanol was gradually added to the prepared mixture while being stirred at room temperature. The hydrolysis-condensation reaction was induced by adding dropwise HCl (1.5 mol) and 1-methylimidazole (5% mol/mol to G, i.e., 0.05 mol). The final mixture was vigorously agitated for 24 h at room temperature. 

### 4.3. Fabrication of Superhydrophobic Fabric by Dip-Pad-Dry-Cure Method

**Figure 10:**
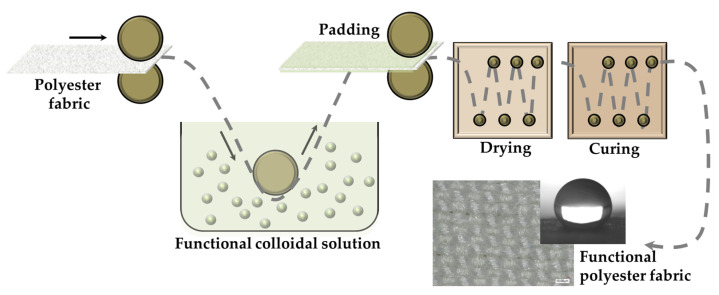
Dip-pad-dry-cure process for the functionalization of polyester fabrics with the opportune functional nanohybrid sol-gel coatings.

The dip-pad-dry-cure procedure was used to impregnate 15 × 15 cm squares of polyester fabrics, as shown in Figure 10. 

The polyester fabric specimens were first submerged in the functional sols for 5 min at room temperature and padded using an automatic padder (basic two roller lab-padder of Mathis, Oberhasli, Switzerland) at a nip pressure of 2 kg/cm^2^ to achieve about 70% of wet gain. 

**Figure 11:**
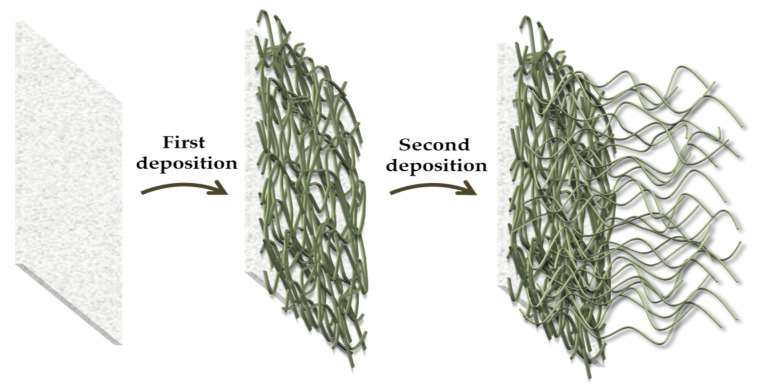
Schematization of the double surface treatment of polyester fabrics.

After drying at 80 °C for a duration of 6 min, the step was repeated a total of three times and the padded fabrics were finally put in oven and cured at 130 °C for 6 min, in a convection oven. Based on an initial study, the best curing conditions (temperature and time) for polyester fabric were selected in order to avoid the thermal degradation of polymers and to achieve the siloxane cross-linking degree typical for robust xerogel coating. Additionally, polyester fabrics were immersed for 5 min in an alkyl(trialkoxy)silane-based ethanol solution (1.0 g, 30 mL), resulting in the deposition of the double-treated specimens (Figure 11).

Finally, the specimens were dried in the oven until constant weight at 130 °C for approximately 5 min. The fabrics were then climatized for 24 h in a typical climate chamber and subsequently weighted.

The total dry-solid add-on was measured through the weight gain (*A wt*.%), by weighing every sample before (*W*_1_) and after being impregnated with the functional formulation and after being subjected to an additional thermal treatment (*W*_2_) and calculated using Equation (1).

(1) $$A\;wt.\;\%=\frac{W_{2}-W_{1}}{W_{2}}\times 100\%$$

The collected add-on% data with the respective code are listed in Table 5 as well as each sample code (where PL was the chosen polyester textile support, G was the GPTMS precursor and C2, C8 and C16 the different long-alkyl chain alkoxysilanes).

### 4.4. Characterizations 

Aqueous liquid repellency: AATCC test method 193-2007 was used to develop a test reagent for the water/alcohol solution tests. 

The static water contact angles (WCA) were measured using the sessile drop technique (in accordance with the international standard ASTM D7334) using a 5 µL water droplet at room temperature. The average of ten readings was used to create one representative WCA.

The spray testing was carried out to assess the dynamic wettability of the treated specimens using the AATCC Test Method 22-2005, appropriate to any textile material substrate. For the spray testing, three fabric samples 150 mm × 150 mm in size were needed to produce one representative standard. A sample of polyester was sprayed with 250 mL of distilled water that has been poured into the tester’s funnel at a 45° angle. The sample was knocked three times before being taken out. The degree of the wetting was evaluated using the water repellency rating (WRR). By evaluating the wetted sample’s appearance with the wetted standard polyester sample, the measurement was elaborated. A higher value indicates greater hydrophobicity.

A scanning electron microscope (SEM, SU-70, Hitachi, Chiyoda, Tokyo, Japan) was used to study the morphology fiber surface. In particular, the pristine and treated polyester fabrics were observed at 2.0 kV and a magnification of 1000 and 4000 for the insets. Prior to testing, gold was sputter-coated onto each sample.

Several water solutions, such as of coffee, milk, tea, methylene blue dyed water, pH = 1 acid (HCl), pH = 14 alkali (NaOH), and salt solution (NaCl), were separately applied to the G_C16_C16 modified polyester fibers in order to assess the wetting behavior. The self-cleaning properties of the functionalized polyester fibers were evaluated by applying soil to their surface and washing it with blue-dyed water.

All of the polyester fabric samples’ ability to carry moisture was assessed using the KERN DBS moisture meter (KERN & SOHN GmbH-TYPE DBS60-3). The thermogravimetry approach was used to assess the moisture-transfer capabilities of each polyester fabric specimen. Each substrate was weighed before and after drying in this procedure to ascertain the variation in moisture. The radiation in the KERN DBS moisture meter passes through the specimen and was converted to thermal energy, which causes the sample to heat up from the inside out. The sample does reflect some radiation, but it does so more strongly in dark samples than in light ones. Since bright samples reflect more thermal radiation than dark ones, they need to be dried at a higher temperature. For this reason, the analysis was performed using a drying temperature of 130 °C and a moisture measurement protocol (the drying method was TIME, drying temperature: 130 °C, unit denoting the result: *M*/*W*). Equation (2) was employed to compute the hygroscopicity ratio, which served as a scale for assessing the hygroscopicity of the different polyester specimens. In the following Equation (2), *M_B_* represents the weight of the conditioned specimen and *M_A_* the starting weight of pristine sample.

(2) $$Hygroscopicity\;ratio\;\%=\frac{M_{B}-M_{A}}{W_{B}}\times 100\%$$

In agreement with ASTM D737-96 standard test procedure, the air permeability of the samples was assessed using a device (FX3300, Tex Test AG, Schwerzenbach, Switzerland) and 125 Pa of air pressure.

## Figures and Tables

**Table 1:** Aqueous liquid repellency data for water/alcohol solution standard test liquids.

Sample Code	Aqueous Solution Repellency Grade Number	Composition (by Volume)	Surface Tensions (Dynes/cm at 25 °C)
PL_G	2	95:5/Water:isopropyl alcohol	50.0
PL_G_C2_ C2	3	90:10/Water:isopropyl alcohol	42.0
PL_G_C8_ C8	3	90:10/Water:isopropyl alcohol	42.0
PL_G_C16_ C16	4	80:20/Water:isopropyl alcohol	33.0
PL_G_C2_ C16	3	90:10/Water:isopropyl alcohol	42.0
PL_G_C8_ C16	3	90:10/Water:isopropyl alcohol	42.0

**Table 2:** Comparison of hydrophobic polyester treatments and achieved WCA values.

Coating System	Coated Fabric	Deposition Method	WCA/°	Ref.
Organophilic graphene nanosheets	Knit polyester	Dip-coating	148	[47]
Superhydrophobic precipitated calcium carbonate	89% polyester: 11% elasthane	Dip-coating	150.2	[48]
Vinyl acetate versatic ester/paraffin wax and sodium chloride	Polyester	Electron beam irradiation	111	[49]
Poly(glycidyl methacrylate) and benzotriazole	PET-co-CEPPA	Surface-initiated PET RAFT ^1^	132	[50]
Ammonium hydroxide and ZnO-Zn(OH)_2_ nanoparticles	Polyester	Dip-coating	138	[51]
TTIP, TEOS and HDTMS	Polyester	Padding	145	[52]
GPTMS, C2, C8 and C16	Polyester	Dip-pad-dry-cure	150.2	[This work]

^1^ surface-initiated photoinduced electron transfer-reversible addition-fragmentation chain transfer polymerization; TTIP = titanium(IV)iso-proproxide, TEOS = tetraethoxysilane, HDTMS = hexadecyltrimethoxysilane.

**Table 3:** Wettability values definite in the spray tests’ ATTCC 22 standards.

Sample Name	Wettability Level	Water Features
PL_G	50 (ISO 1)	Complete wetting of the entire specimen face
PL_G_C2_C2	50 (ISO 1)	Complete wetting of the entire specimen face
PL_G_C8_C8	100 (ISO 5)	No wetting of the specimen face
PL_G_C16_C16	100 (ISO 5)	No wetting of the specimen face
PL_G_C2_C16	100 (ISO 5)	No wetting of the specimen face
PL_G_C8_C16	100 (ISO 5)	No wetting of the specimen face

**Table 4:** Data for standard test of moisture adsorption of the different pristine and coated polyester samples.

Sample Code	Weight (g)	Drying Temperature (°C)	Drying Time (s) ^1^	Humidity (%)
PL	1.189	130	54	1.26
PL_G	1.571	130	51	0.76
PL_G_C2_C2	1.645	130	52	1.03
PL_G_C8_C8	1.609	130	51	1.06
PL_G_C16_C16	1.693	130	51	1.00
PL_G_C2_C16	1.564	130	50	1.02
PL_G_C8_C16	1.545	130	48	0.97

^1^ drying time until constant weight

**Table 5:** Sample codes (showing coatings compositions), total add on values and sample appearance of the polyester fabrics treated with the different functional nanohybrid sol-gel coatings.

Sample Code	First Deposition	Second Deposition	Total Add on wt. % (A)	Sample Appearance
PL_G	G	–	0.9	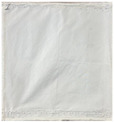
PL_G_C2_C2	G and C2	C2	2.16	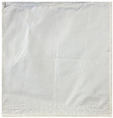
PL_G_C8_C8	G and C8	C8	5.7	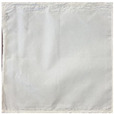
PL_G_C16_C16	G and C16	C16	11.8	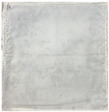
PL_G_C2_C16	G and C2	C16	2.6	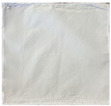
PL_G_C8_C16	G and C8	C16	4.4	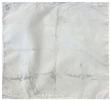

## Data Availability

Not applicable.
